# Numerical Simulation of an Intramedullary Elastic Nail: Expansion Phase and Load-Bearing Behavior

**DOI:** 10.3389/fbioe.2018.00174

**Published:** 2018-11-21

**Authors:** Giulia Pascoletti, Filippo Cianetti, Giovanni Putame, Mara Terzini, Elisabetta M. Zanetti

**Affiliations:** ^1^Department of Engineering, University of Perugia, Perugia, Italy; ^2^Polito^BIO^Med Lab, Department of Mechanical and Aerospace Engineering, Politecnico di Torino, Turin, Italy

**Keywords:** multibody analysis, FE analysis, sliding contacts, intramedullary nails, Marchetti-Vicenzi's nail, flexible bodies

## Abstract

The Marchetti-Vicenzi's nail is an intramedullary device where six curved nails are kept straight by a closing ring in order to allow their insertion into the medullary canal of a long bone; in a following step, these nails stabilize the fracture due to the ring withdrawal and to the consequent elastic expansion of the nails. Pre-clinical testing of this sort of device is strongly advocated in order to be able to foresee their stability inside the medullary canal and to quantify their stiffening action on a broken bone. In this numerical work, an MB (Multi Body) model of the device has been developed, with the dual purpose of evaluating forces between the bone and the system components during its progressive opening and verifying the behavior of the stabilized bone when it undergoes external loading. Different solutions, for flexible body modeling (discretization with lumped parameters, “flexible body,” “FE Part”), have been analyzed and compared in terms of accuracy of results and required computational resources. Contact parameters have been identified and criteria to simplify geometries and therefore to reduce simulation times have been given. Results have allowed to demonstrate how a moderate lateral force is able to dislocate the fracture and how the final position of the retention nut can be optimized. On the whole, a tool for the pre-clinical testing of elastic intramedullary nails has been given.

## Introduction

Intramedullary nails are orthopedic devices for fractures fixation, particularly successful in the treatment of long bones fractures (Eveleigh, 1994; Bong et al., 2007; Dutta and Datta, 2008). The difference from standard “conservative” systems (conservative methods such as plasters, splints, braces) is the partial substitution of bone load bearing function in spite of its full replacement (Müller et al., 1991; Eveleigh, 1994; Bong et al., 2007; Dutta and Datta, 2008). This behavior allows providing a continuous remodeling stimulus to bone, promoting the callus formation and consequently the healing process (Yamaji et al., 2001; Dutta and Datta, 2008).

Internal fixation devices have evolved over time (Court-Brown, 1991; Hessmann, 2015): at the beginning, they were rigid systems composed by one single nail implanted into the medullary cavity (Kuntscher, 1958). Afterwards systems with locking screws have been designed (Kempf et al., 1985; Boero Baroncelli et al., 2013), and finally, flexible nails have been developed, usually inserted at least in pairs into the medullary canal (Pospula and Abu Noor, 2009; Abosala et al., 2011).

**Figure 1:**
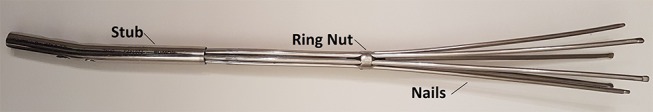
Marchetti-Vicenzi's nail.

The Marchetti-Vicenzi's nail falls within the category of flexible intramedullary systems (Figure 1). This device has been developed for the first time in the middle ‘90s and its design has evolved over the years; its latest version is characterized by the presence of six nails which are pre-curved away from the longitudinal nail axis. These nails are kept closed by a ring nut, working as a retention system, in order to allow their introduction into the medullary canal. Once the device is implanted, this ring is withdrawn and the nails are free to elastically expand into the medullary cavity and stabilize the fracture (Vicenzi, 1994). Clinical studies concerning this device have produced contrasting results. Marchetti-Vicenzi's nail has been proved to allow a rapid recovery of the shoulder mobility in the treatment of humerus diaphyseal fractures (Tennant et al., 2002; Martínez et al., 2004; Zerbinati et al., 2004). However, with reference to femoral fractures, the system does not seem to be able to provide the required stability according to some studies (Anastopoulos et al., 2001; Madan et al., 2003), leading to implant failure or long healing periods.

This research work is aimed to set up a methodology to optimize these devices and to foresee their performance before clinical applications. The design of flexible intramedullary nails is complex due to the presence of bodies undergoing large deformations (the expanding nails) and to a high number of sliding contacts (among nails, between each nail and the nut, and between the nails and the bone). In the following sections, MB modeling of a Marchetti-Vicenzi's nail will be discussed in detail, as well as its performance in terms of its behavior during the insertion phase into the femur and the stabilization provided. A fractured femur has been considered as a benchmark since a further aim of this work is to improve this kind of treatment on femurs in order to use it in the near future.

## Methods

The multibody model has been realized by means of MSC Adams software (v. 17, by MSC Software Corporation). The first step for the generation of the MB model has required identifying which elements, among nails, sliding ring nut and fractured bone, need to be modeled as rigid bodies and which ones should be considered as deformable (flexible) bodies. The ring and the bone exhibit small deformations when compared with deformations of the nails due to their plastic pre-curvature and to their small radius (1.5 mm). Therefore, only nails have been modeled as deformable bodies.

### Modeling deformable nails

**Figure 2:**
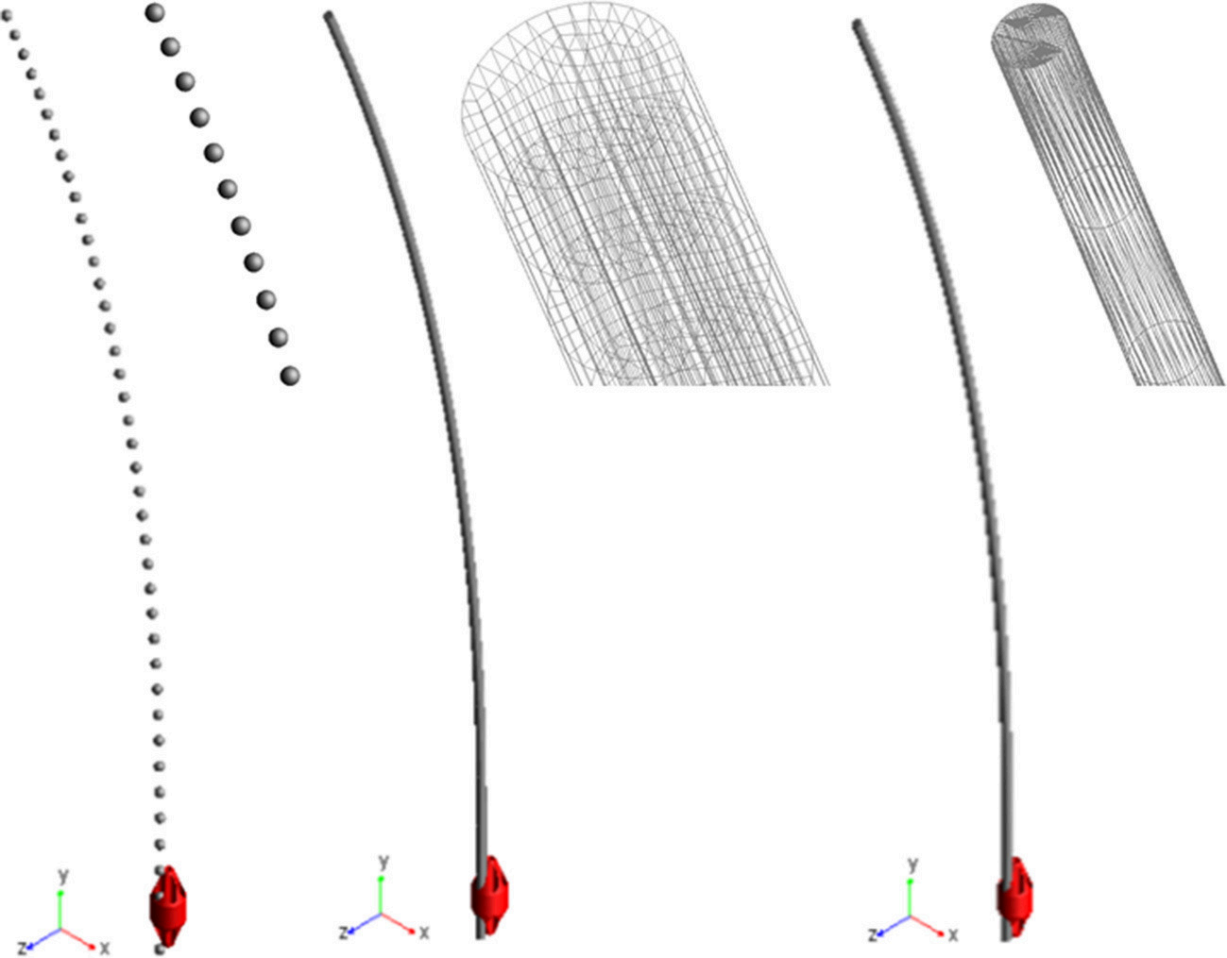
Three modeling solutions for nails. From left to right: lumped mass and stiffness elements, flexible body (MNF), FE Part.

Three different approaches to deformable nails modeling have been compared (Figure 2); in each of them, the nail element has been studied as a three-dimensional slender beam, made of biocompatible stainless steel AISI 316 LVM (E = 2 × 10^11^ MPa, ρ = 8,000 Kg/m^3^ and υ = 0.3). The first method consists of a discretization of the nail through a combination of lumped elements with inertial and stiffness properties. Each element is composed of two rigid spheres, placed at the ends of a massless beam, which produces forces and moments linearly dependent on relative displacements and velocities of the beam's endpoints (MSC Adams, 2000; MSC SimCompanion, 2017c). Inertial properties of the nail are attributed to the rigid spheres in accordance with the lumped mass method; all these elements are arranged in series to reproduce the actual nail geometry. Therefore, the mass is equal to half the mass of the beam connecting two spheres for the first and the last spheres, whereas the mass is twice this value for internal bodies. On the other side, stiffness properties of the beams have been computed from a beam with a circular cross section identical to the nail cross section. The number of rigid bodies for the discretization has been determined imposing that at least two spheres were engaged into the ring's hole (7 mm long), during each step of the sliding phase; as a result, the nail has been discretized by 41 spheres.

The second approach required creating and importing a finite element model of the nail to define a “flexible body.” The geometry has been replicated extruding a circular section, meshed by 100 4-node shell elements; this section was extruded along a spline coincident with the centreline and the final model reproduced the nail's volume through 18600 20-node solid elements (4th order shape functions). The finite element model was imported into Adams as a MNF (Modal Neutral File, Zanetti et al., 2017). As it is based on the Craig-Bampton theory, boundary DOF must be defined (Ansys Inc., 2013; MSC SimCompanion, 2017b); therefore, the nodes placed at the center of the upper and lower nail sections have been selected as interface points.

The last method made use of the so called FE Parts, that are a modeling tool for flexible bodies provided by the multibody software (MSC SimCompanion, 2017a). This tool allows generating both straight and curved 3D beams: a spline was defined that was a guide curve for the centreline, along with the shape and the size of the beam section and material properties. The final model was a beam, meshed by a set of about 2570 tetrahedric solid elements (they are based on a non-linear finite element formulation, used in flexible multibody dynamics to describe large deformation of moving bodies, but no further details are disclosed by the software producer).

### Ring nut modeling

The ring nut is a key element, governing the contact forces driving the device performance.

**Figure 3:**
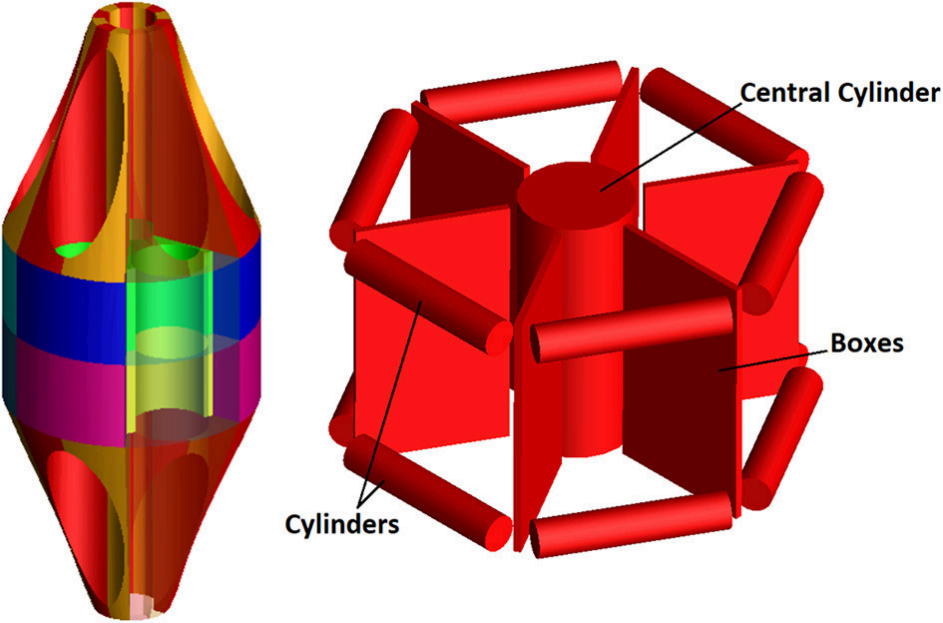
Ring Models: segmented ring **(Left);** “conceptual” ring **(Right)**.

The direct importation into the model of the 3D ring geometry as a rigid body leads to the difficult calculations of contact forces between two coaxial cylinders (Webex, 2012). As a first step, the full ring geometry has been therefore segmented into smaller elements in order to reduce the contact detection area (see differently colored volumes in Figure 3). In addition, a “conceptual” model of the ring nut has been created in order to further simplify numerical simulations and reduce computation time. This model simulates the central cylindrical part of the nut and is reported in Figure 3. The advantage of this solution compared to the segmented ring model is that localized contacts take the place of distributed contacts and the number of contacts per nail is reduced. This simplified model has been validated comparing its results to the results obtained using the original geometry (section Contact Parameters Tuning).

### Fractured femur modeling

The fractured bone has been modeled as a solid body, through the importation of the 3D femur geometry into the simulation environment (Zanetti and Bignardi, 2009). Only the cortical bone has been modeled in order to obtain a conservative estimate: the trabecular bone is very thin inside the medullary canal and it could be easily fractured by the nails; a fracture along a plane inclined of 30° [type 32-A2, according to AO classification (Femoral shaft - Diagnosis - AO Surgery Reference)^1^ has been simulated. The initial relative position of the fractured bone parts has been governed through a massless element, oriented as the fracture plane, with linear stiffness and damping properties (MSC SimCompanion, 2017c).

### System's constraints

One end of all nails has been fully constrained by a fixed joint, in order to simulate the stub (Figure 1). The ring slides along the vertical direction (*y* axis in Figure 2) by means of a translational joint, following a law of motion defined by a quintic polynomial (“*STEP5”* function). The displacement law has been imposed so that the ring covers 33 cm within 5 s, both throughout the upward and the downward motion. The two bone's fragments have been completely constrained by two fixed joints applied at their center of mass; these boundary conditions allow removing bone displacements and rotations during the first two phases of simulation, approximating actual conditions in surgery.

### Contact parameters

All contact forces have been simulated, making use of *IMPACT* formulation in Adams.

This formulation includes an elastic and a damping component, defined through four parameters (*K*, *e*, *C*_max_, and *d*_max_), as detailed in the following:

(1) $$\begin{array}{r} F_{n}=K\cdot g^{e}+STEP\left(g,0,0,d_{\max},C_{\max}\right)\cdot dg/dt \end{array}$$

where *g* is a parameter that represents the penetration between the involved geometries, *dg/dt* is the penetration velocity evaluated at the contact point, *e* is the elastic force exponent, *d*_*max*_ is the penetration depth and *C*_*max*_ is the damping coefficient. The *STEP* function imposes a gradual variation of the damping coefficient, according to the following cubic polynomial law:

(2) $$STEP(x,x_{0},h_{0},x_{1},h_{1})\text{​}=\text{​}\{\begin{array}{cc} h_{0\;}\; & if\;x\leq x_{0}\\ h_{0}\text{​}+\text{​}a\cdot \Delta^{2}\left(3-2\Delta \right)\; & \;if\;x_{0}\text{​}<\text{​}x\text{​}<\text{​}x_{1}\\ h_{1}\; & if\;x\geq x_{1} \end{array}$$

where *x*_0_ and *x*_1_ are limit input values of the *STEP* function; *h*_0_ and *h*_1_ are the initial and final function values; *a* = *h*_0_ *- h*_1_ and Δ = (*x*- *x*_0_)/(*x*_1_ - *x*_0_).

All these parameters have been initially estimated taking into consideration the geometry and materials involved in the model; subsequently, they have been tuned examining results of a set of simulation tests: the “ideal” set of parameters should allow performing simulations in reasonable times, avoiding excessive penetration among objects.

All simulations have been performed with frictionless contacts between the nails and the medullary canal; this is a conservative hypothesis, since friction would have a stabilizing action, limiting movements of the nails inside the bone during the loading phase.

### FE model for results validation

**Figure 4:**
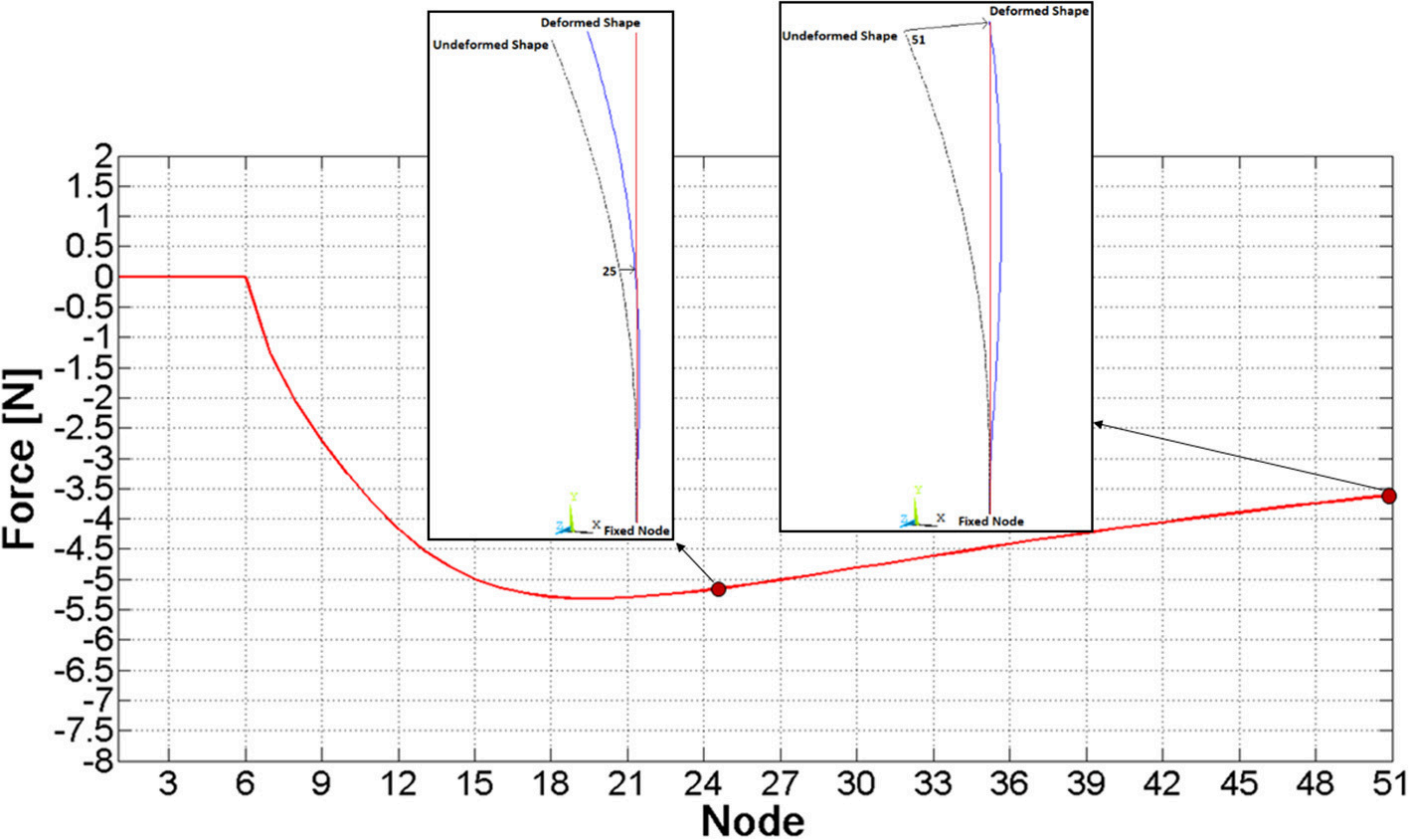
Pattern of the force required to move a given node to the vertical axis.

A simple finite element model of a single nail has been created (by Ansys v. 17) as a benchmark to check MB model results. Nail's centreline has been replicated through a spline, meshed by means of 50 beam elements. All six DOF of the base node (“fixed node” in Figure 4) have been constrained, and sequential analyses have been performed, where, node by node, a *z* displacement equal to the node's *z* coordinate itself (Figure 4) has been imposed (that is the examined node has been brought to the vertical axis) and the respective constraining force has been measured.

As a result of this analysis, the force required to bring each node to the zero *z* coordinate was assessed and it has been plotted in Figure 4; the force magnitude initially grows, it reaches a maximum value on node 20th (about 5 N), and finally it decreases.

## Results and discussion

### Choice of the best modeling approach

**Figure 5:**
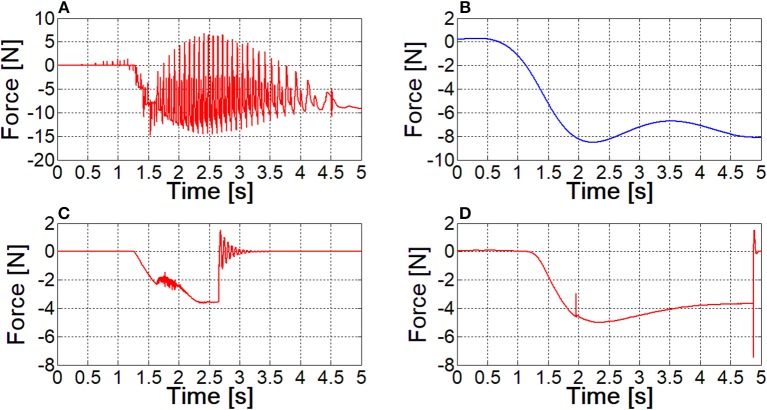
The contact force between the nail and the sliding ring, as resulting from different models; **(A)** lumped parameters model; **(B)** filtered curve of lumped parameters model result; **(C)** flexible body model; **(D)** FE Part model.

Comparisons among the three nail models have been performed analyzing the interaction of a single nail with the ring. At this stage, contact parameters have been set equal to their default values. The discretized nail model has required implementing 41·8 contacts (since the spheres are 41 and the segments composing each nail guide in the ring are 8), whereas 8 contact couples were sufficient for the Flexible Body and the FE Part models. Each model has been validated analyzing the pattern of *z* force component at the nail's constraint (Figure 5), which had to replicate the force behavior reported in Figure 4. Obtained results are shown in the Figure 5.

The contact force for the lumped parameters model of the nail exhibits many peaks, corresponding to spheres coming in and out of contact within the ring hole. The blue curve in Figure 5B can be obtained with a filtering operation (a second order, low pass Butterworth filter, with a cut-off frequency of about 1 Hz has been applied to the signal), and its pattern is similar to the one depicted in Figure 4, however absolute values are quite different.

In Figure 5C, the sudden change of the force amplitude at 2.5 s time instant, along with the consequent vibrations are a clear sign that the Flexible Body model is not able to keep the nail in contact with the ring: there is a sudden compenetration and since that time instant the contact boundary is over.

With reference to the FE Part model, the curve is smooth and its pattern and amplitude are comparable to those obtained from FE model; again, compenetration takes place as in the Flexible Body simulation, however it occurs at the final phase of the ring movement.

Simulation times of the three formulations are 3.4, 6.5, and 5.3 min respectively. On the basis of these results and of further similar tests, the Flexible Body model has been discarded, because of the higher computational times and because the contact management is more critical. FE Part formulation has proved to be sufficiently robust to obtain an accurate contact force curve even when using default contact parameters values, leaving a wide margin for their tuning. Having considered this aspect and the lower number of required contacts, the FE Part model has been established to be the best approach, in spite of its higher simulation time compared to the lumped parameters model.

### Contact parameters tuning

A gradual tuning process of contact parameters has been performed; as described above, the only contact between a single nail and the ring nut has been modeled as a first step; after, a second nail has been added as well as the contact between two adjacent FE Parts; finally, the complete system has been considered. Table 1 shows the best set of parameters obtained for each model, and images illustrate the respective configurations at the final stage of ring nut movement.

**Table 1:** Contact parameters for the real ring geometry.

		**Contact**	***K* [N/m^e^]**	***e***	***C* [Ns/m]**	***d* [m]**
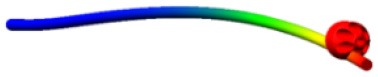	Single Nail Model	Nail to Ring	2E+08	2.2	1E+04	1E-04
		Nail to Nail	–	–	–	–
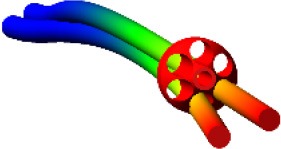	Two Nails Model	Nail to Ring	5E+08	2.2	1E+04	1E-04
		Nail to Nail	1E+08	2.2	1E+03	1E-04
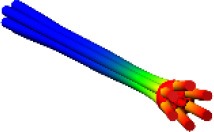	Six Nails Model	Nail to Ring	4E+08	2.2	1E+04	1E-04
		Nail to Nail	1E+08	2.2	1E+04	1E-04

**Figure 6:**
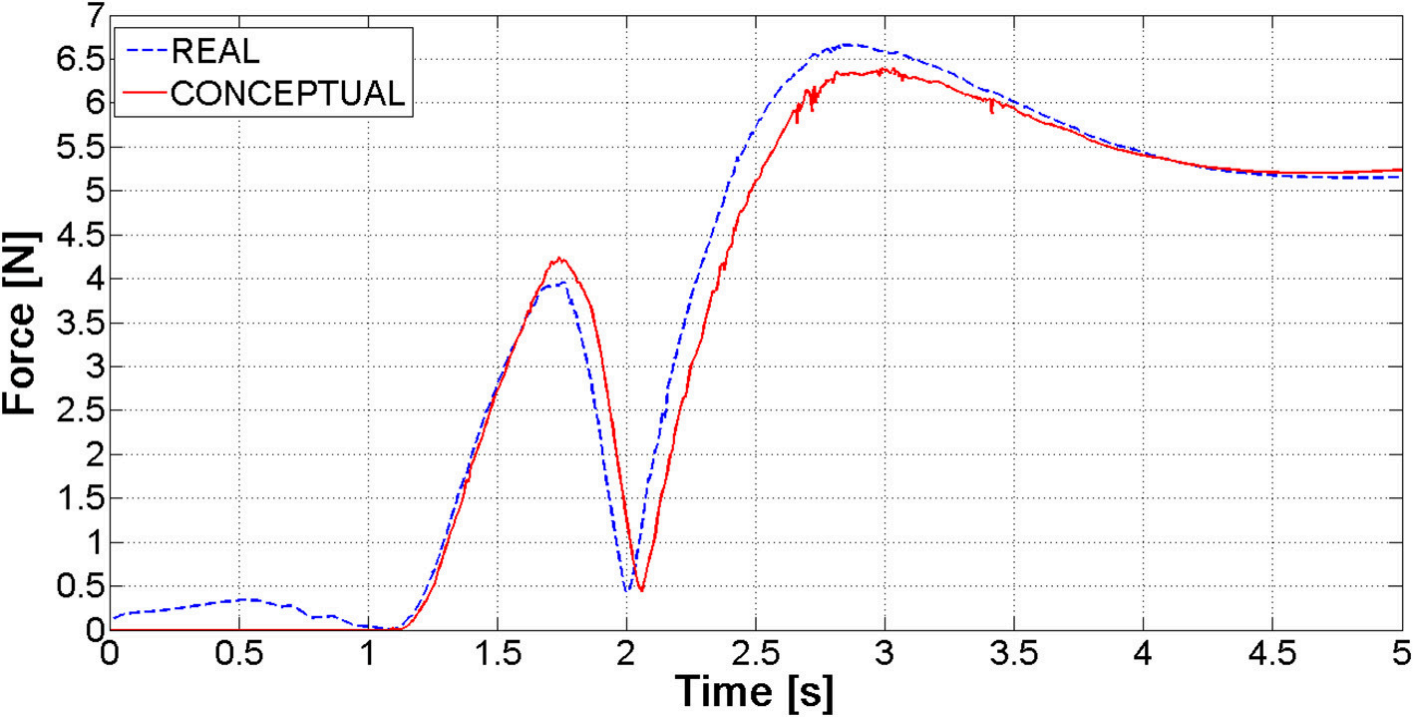
Ring constraining force for the real and the conceptual ring.

The tuning process has demonstrated that “optimal” contact parameters may change in relation to the number of elements (and consequently the expected contact force), even with reference to the same contact couple (i.e., nail-to-ring or nail-to-nail). Once parameters for the six nails-real ring model have been established, the “simplified” ring has been validated. Figure 6 shows the magnitude of the force at the bounded end of one of the nails, for the actual and the simplified ring: there is a good match between the two curves, the maximum difference between peak values reaching 4%.

**Figure 7:**
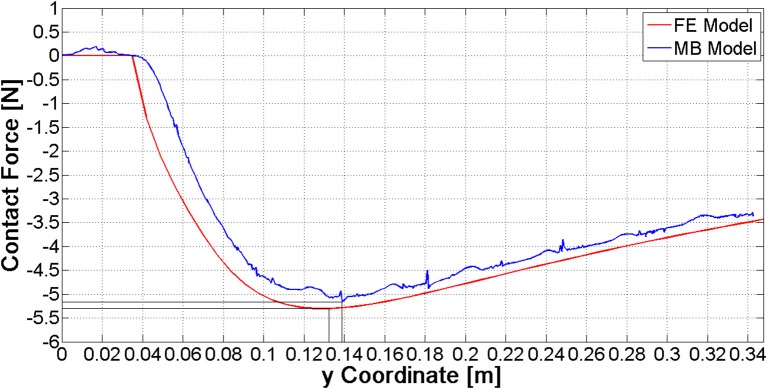
Comparison of contact force curves between the FE and the MB model.

The single nail model has been validated comparing the ring-to-nail force with the force computed from the FE model (Figure 7). The comparison has showed that these patterns are very close, with a deviation of the MB peak value (5.16 N) equal to about 3% compared to the FE peak value (5.30 N). Besides, the location of this peak value along the nail (abscissa in Figure 7) is the same.

The simplified ring has allowed a further optimization of contact parameters in order to limit compenetration phenomena (Table 2); this was made possible by the reduction of the model complexity: with the original nut model, parameters reported in Table 2 would have led to unacceptable simulation times. In fact, using the internal solver setting and the real ring geometry model, the simulation calculates around one half of the ring path in 2 h and, from that point on, it cannot proceed due to high penetration phenomena between the nails and the ring. In the same conditions, the simulation with the simplified ring geometry is completed in 50 min (simulations have been performed on laptop equipped with a i5 processor and 12 GB RAM).

**Table 2:** Contact parameters for the conceptual ring geometry.

**Contact**	***K* [N/m^e^]**	***e***	***C* [Ns/m]**	***d* [m]**
Nail to Central Cylinder	4E+08	2.2	1E+04	1E-04
Nail to Cylinders	4E+08	2.2	1E+04	1E-06
Nail to Boxes	1E+10	2.2	1E+03	1E-06
Nail to Nail	2E+09	2.2	1E+04	1E-05

### Interaction between the device and the medullary canal

**Figure 8:**
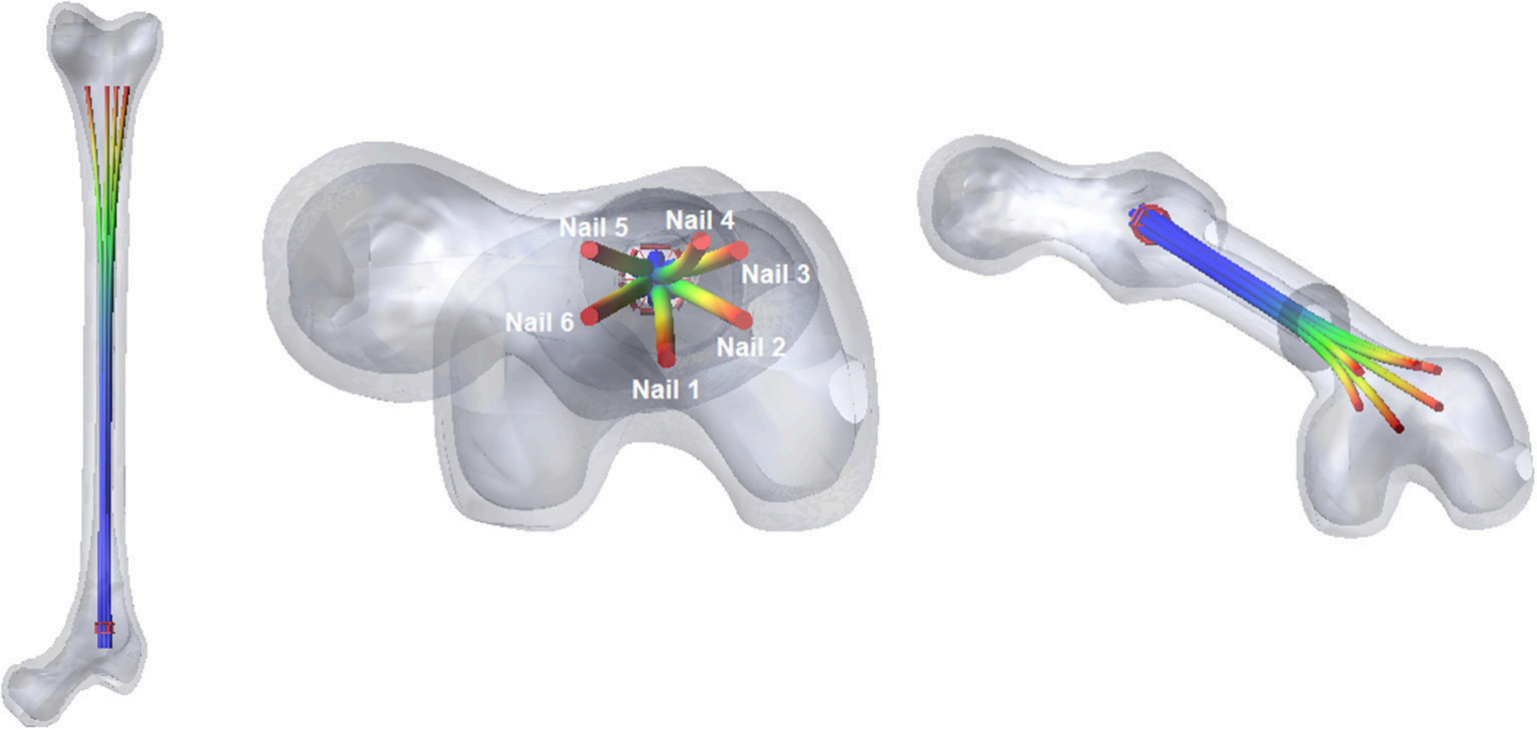
The fully open configuration of the nail into the medullary canal.

The two bone's fragments have been constrained by two fixed joints, and standard contact parameters have been set, to study the contact between the nails and the inner wall of the medullary cavity. When the device is fully open into the femur (Figure 8), the nails adapt their shape to the bone cavity.

**Figure 9:**
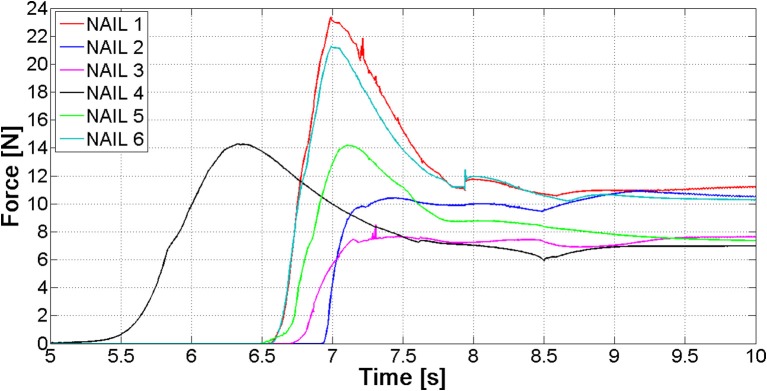
Contact forces between the six nails and the distal fragment.

The most relevant forces are those between the nails and the distal femur fragment. These forces are shown in Figure 9; they are different for each nail, because the geometry of the canal is not axial-symmetric.

The values of forces at the end of the opening phase are produced not only by the action of the distributed contact along the medullary canal, but also by the forces that nails exert on one another (Figure 8). This observation explains why the final force values of curves reported in Figure 9 stand between 7 and 12 N, while both FE and MB single nail models output was equal to 4 N (Figure 7).

**Figure 10:**
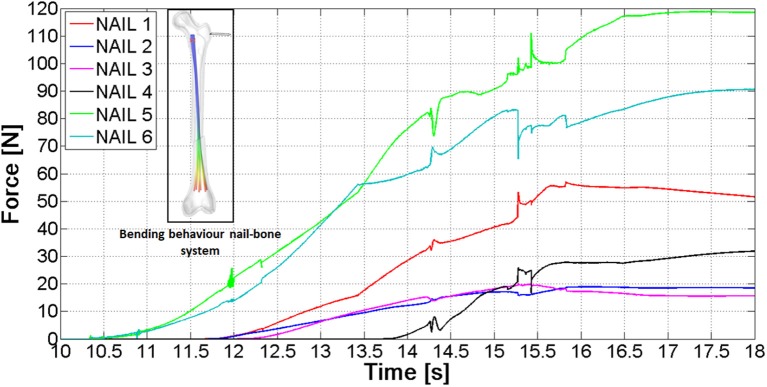
Contact forces between the nails and the proximal bone fragment.

After the implantation of the Marchetti-Vicenzi nail, the fractured femur has been loaded: the proximal fragment has been subjected to a growing quasi-static horizontal force [ranging from 0 to 200 N with a quintic polynomial step law (Terzini et al., 2017), see contoured image in Figure 10]. The forces between the six nails and the proximal bone fragment throughout the loading phase are depicted in Figure 10: the nails start contacting the proximal bone at time 10 s, and from that moment on, contact forces rise.

The final contact force ranges between 15 and 118 N; these different values are due to the fact that nails come into contact with different portions of the proximal fragment.

**Figure 11:**
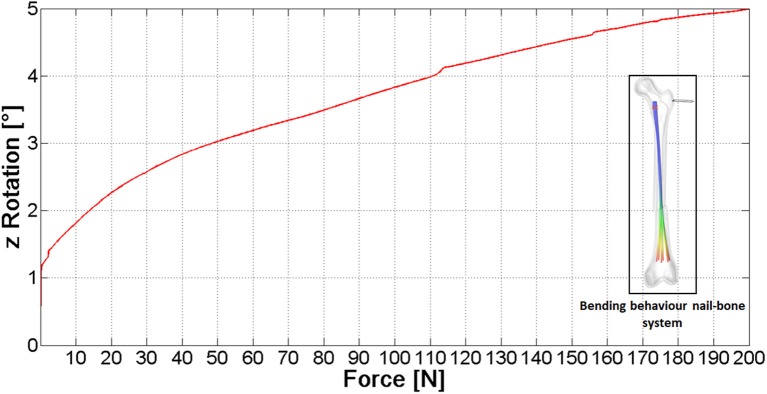
The pattern of rotation vs. the applied external force.

Figure 11 shows the relative rotation between the bone fragments vs. the external load (see the arrow in contoured image in Figure 11). The curve here reported proves the device capability to stiffen the broken femur toward bending loads: if the device would not be present, the slope of this curve would be theoretically infinite. The video provided in the Supplementary Material section shows the full simulation.

These data allow to reach a conservative estimate since they have been obtained without taking into account the friction between nails and bone, the contact force and friction between the two bone fragments and without modeling the porous spongy component; therefore the actual femoral rotation is expected to be lower. According to these results, the nail action is relevant, however a moderate (200 N) lateral force applied on a standing subject (fixed foot) would be able to significantly dislocate the fracture. This could be the reason why the performance of this fixation device has been proved to be poor on the femoral bone (Anastopoulos et al., 2001; Madan et al., 2003), where acting forces are higher compared to upper extremity bones, on average.

The authors have here chosen to follow a “global” approach, considering the nail stiffening action: this action is strictly related to relative displacement at the fracture site since the larger is this action the lesser relative displacements are. The reason why more localized measurements (such as the above cited relative displacements) have not been here considered is the respective dependency on boundary conditions, such as the geometry of the fracture rim (i.e., transverse or oblique) and on friction between contacting surfaces. This would hamper obtaining general results on the performance of a given bone-nail system. Indeed, a patient-specific planning would require performing such evaluations since healing is strictly related to displacements at the fracture site.

**Figure 12:**
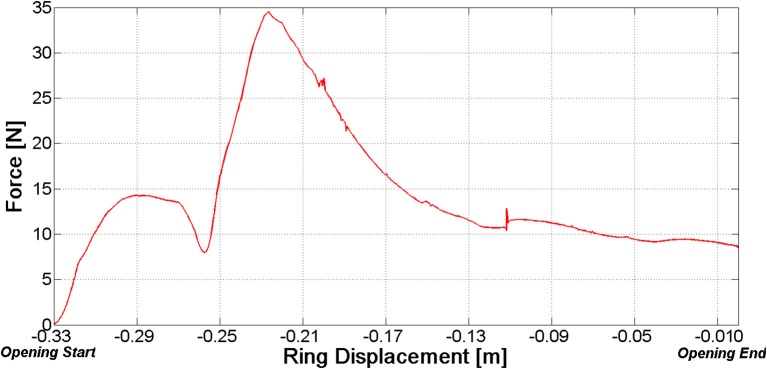
Net force on the distal fragment vs. ring position (opening phase).

However, it should be stressed that there is still a margin to improve. Figure 12 reports the net force at the distal fragment vs. the ring position. With reference to the opening phase, this force reaches a peak value before the ring has been completely withdrawn. As a consequence, the most stable configuration of the fractured bone—nail system is not the completely open configuration.

**Figure 13:**
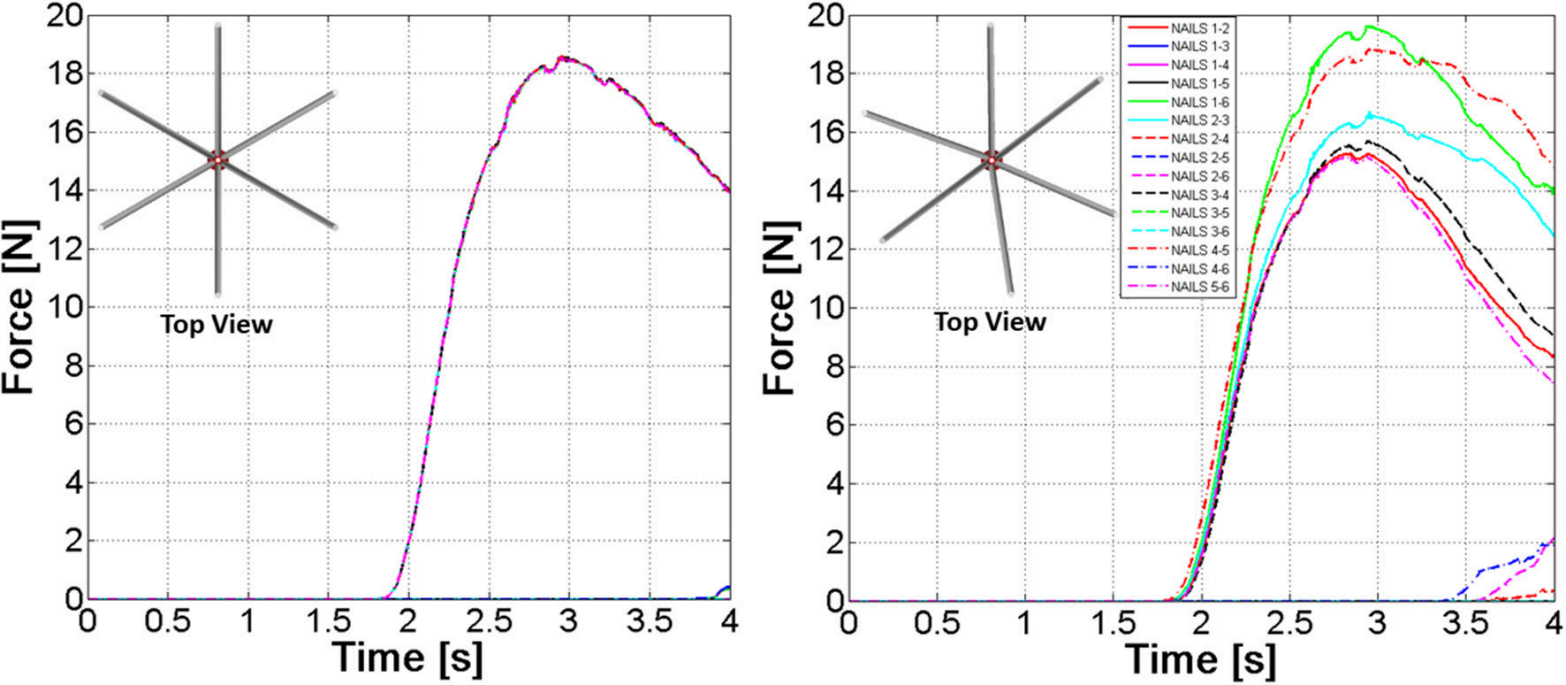
Nail-to-nail contact forces. “Ideal” device **(Left)**—“Realistic” device **(Right)**.

The model described in the previous sections was based on the hypothesis of an ideal assembly of the nails; that is nails centrelines have been placed at exactly 60° steps. A further model has been developed where an angular position error ranging between 0° and 10° has been randomly assigned to each nail. Figure 13 clearly outlines differences between FE Part contact curves in the “ideal” nails system or in the “realistic” one. Differences in contact forces can reach 50%, therefore the employment of a probabilistic design is strongly advocated for this sort of devices.

## Conclusions

A numerical model for the pre-clinical study of a flexible nail system for bone fracture synthesis has been here set up. The complexity of this work derives from solid bodies contacting flexible bodies undergoing large displacements. The final aim was to provide an efficient tool to optimize the design of these devices; simulation complexity had to be limited in order to make possible studying many different solutions in reasonable times. A multibody model including flexible elements modeled as “FE Parts” has resulted to fit the aim, as long as key geometries are simplified in order to reduce computational costs. More in detail, “undetermined” contact between congruent surfaces should be avoided in favor of localized contacts.

The setup model is able to reproduce the Marchetti-Vicenzi nails closure, its opening inside the medullary canal and its response to bending. The single nail behavior during the opening phase has been validated against the results of a finite element model reproducing a curved beam, subjected to an imposed displacement.

The ability of the device to stiffen the broken bone behavior has been proved and quantified, through the analysis of force/displacement curves. The three stages of the analysis (closure-opening-external loading) can be now simulated within2 h, while it required more than 20 h prior to model optimization.

In addition, the results of the simulation can be a support to improve the device's performance. For example, it was here demonstrated how the final ring position can be optimized analyzing the pattern of forces between the medullary canal and nails during the elastic expansion of the nails.

The model has been also applied to study eventual non-ideal assemblies of the nails, demonstrating how a probabilistic approach to the design of such devices is highly recommendable.

This tool will be used to optimize nails geometry (curvature, length, etc.) and its final configuration (ring position), in relation to different shapes of the femoral canal, since it can provide indications about the maximum loads which can be withstood without incurring into significant fracture dislocations.

## Author contributions

GPa, GPu, and MT have set up the numerical models, have performed numerical simulations, and have worked on parameters optimization. GPa was specifically involved in the analysis of different modeling solutions, and on the set up of simplified geometries. FC and EZ have supervised the work, giving hints for simulation times reduction and for model validation. EZ has specifically addressed biomechanical aspects and has organized the work and its objectives. The paper has been co-written by all the authors.

### Conflict of interest statement

The authors declare that the research was conducted in the absence of any commercial or financial relationships that could be construed as a potential conflict of interest.

## Footnotes

^1^Available at: https://www2.aofoundation.org/wps/portal/surgery
