# Supplementary material for: Numerical Simulation of an Intramedullary Elastic Nail: Expansion Phase and Load-Bearing Behavior
Source: Front Bioeng Biotechnol. 2018 Nov 21;6:174. doi: 10.3389/fbioe.2018.00174 (PMC6258715; doi:10.3389/fbioe.2018.00174)
Supplement: Supplementary file 1 [file Table_1.DOCX]

Supplementary Material

Numerical Simulation of the Behaviour of an Intramedullary Elastic Nail

**G. Pascoletti, F. Cianetti, G. Putame, M. Terzini, E.M. Zanetti^*^**

*** Correspondence:** Elisabetta M. Zanetti: elisabetta.zanetti@unipg.it

# Supplementary Data – Video of the Simulation

This video shows the multibody model of an orthopaedical device for femur fractures stabilization. The animation concerns the device closure and its opening inside the medullary canal and the bone-nail system response during femur loading. The video presents also the comparison between the behaviour of the multibody model and the real device throughout the closure phase.
